# A self-directed online resource to enhance coach learning: a 21st-century approach to coach education

**DOI:** 10.3389/fspor.2025.1588636

**Published:** 2025-09-22

**Authors:** Koon Teck Koh, Fernando Santos, Tarkington J. Newman

**Affiliations:** ^1^ National Institute of Education, Physical Education & Sports Science, Nanyang Technological University, Singapore, Singapore; ^2^ Centro de Investigação e Inovação em Educação, inED, Escola Superior de Educação, Instituto Politécnico do Porto, Porto, Portugal; ^3^ Sport Social Work Research Lab, College of Social Work, University of Kentucky, Lexington, KY, United States

**Keywords:** pedagogy, technology, mobile application, life skills, values

## Abstract

**Introduction:**

Physical education and sport settings provide dynamic environments conducive to learning values and life skills. However, not every programme that aims to promote healthy youth development has been successful. Such ineffectiveness is often due, in part, to a lack of requisite knowledge and efficacy among coaches. Nonetheless, preliminary research has begun highlighting the potential of online self-directed resources, a contemporary 21st-century approach to coach education and development. The present study explored the development of a coach observation tool (COT) mobile application to support coaches' learning and the participants' perceptions and recommendations of COT for future enhancement.

**Methods:**

A total of 34 coach participants from team and individual sports were involved in the present study. Two 60 min lessons were recorded from each participant. The participants were also interviewed individually after the second observation. The lesson plans submitted by the participants were used to link the data generated by the COT mobile application with the percentage of time spent on sports skills, values and life skill development. The COT data were analysed using a paired-sample *t*-test, while thematic analysis was employed to examine the interview transcripts.

**Results & discussion:**

Findings indicate that i) key features of the COT are appropriate and practical for coaches; ii) the use of the COT supported self-reflection among coaches, particularly related to facilitative coaching practices and an increased awareness in the designing and delivery of programming. However, additional application features (e.g., incorporating athlete feedback, using Artificial Intelligence for data management) may further enhance the potential to support coach learning effectively. Finally, the theoretical and practical implications for coaches, administrators, and coach educators are discussed.

## 1 Introduction

Youth sports coaches and other youth sports leaders (e.g., physical education teachers, facilitators, camp counsellors) (1) serve a crucial role within the youth sport system (2) and are key contributors to the healthy development of youth (3). Yet, throughout much of the coaching science scholarship, the definition of an “effective” leader remained elusive, with conceptualisations originating from sports management [e.g., (4)], kinesiology [e.g., (5)], and sport psychology (6). In response to this lack of clarity, Côtè and Gilbert (7) proposed the *Coaching Model*, which denotes the underlying aspects of coaching effectiveness and expertise through three key components: coach knowledge, athlete outcomes, and coaching contexts. *Coach knowledge* encompasses professional, interpersonal, and intrapersonal knowledge and highlights the importance of teaching sport-specific skills, developing relationships with others, and being introspective and reflective. *Athlete outcomes* refer to the development of competence, confidence, connection, and character/caring among youth athletes and, in turn, how effective coaches support holistic athlete development. In contemporary youth sports scholarship [e.g., (8, 9)], key developmental outcomes commonly include values (e.g., embracing difference) and life skills (e.g., teamwork). Within the model, *coaching contexts* refer to the unique settings in which coaches coach, lead, and teach athletes. In the end, Côtè and Gilbert (7) defined coaching effectiveness as “the consistent application of integrated professional, interpersonal, and intrapersonal knowledge to improve athletes' competence, confidence, connection, and character in specific coaching contexts.” (p. 316).

Indeed, one of the hallmarks of effective coaches is a desire to support athletes' healthy, holistic development. To do this, coaches often aim to promote positive youth development (PYD) by utilising indirect (e.g., designing structured activities) and direct (e.g., debriefing learning experiences) facilitative coaching strategies to intentionally teach transferable values and life skills (10, 11). Values are recognised as “the principles and fundamental convictions, which act as general guides to behaviour, the standards by which particular actions are judged to be good or desirable” (12). Similarly, life skills are defined as “those internal personal assets, characteristics and skills such as goal setting, emotional control, self-esteem, and hard work ethic that can be facilitated or developed in sport and are transferred for use in non-sport settings” (13). The aim to foster the development of transferable values and life skills must be central to a coach's philosophy and, therefore, is often a foundational component of one's own social identity and belief system (14). Thus, values and life skills, which are inherently important to a coach, are reflected in their coaching pedagogy and praxis.

Coaches have several avenues through which they can learn how to be effective, particularly when aiming to facilitate the development of values and life skills. From an experiential learning theory perspective [see (15)], learners (e.g., coaches) gain perspective, construct knowledge, and develop skills through their own firsthand experiences upon which they engage in a process of self-reflection (16). To this end, Werthner and Trudel (17) proposed meditated, unmediated, and internal learning situations that serve as unique learning opportunities. In mediated learning situations (e.g., educational courses, formal mentoring), learning materials and content are decided upon and directed by a person other than the learner (e.g., educator, facilitator). Conversely, in unmediated learning situations, there is no formal instructor; rather, the learner is responsible for choosing what to learn. Finally, in internal learning situations, the learner is not exposed to novel content; instead, the learner reconsiders, reflects, and reevaluates their existing knowledge and practices.

However, the effectiveness of these different learning situations has yielded mixed results. For example, mediated learning situations—such as formal coach education programmes, which aim to develop coaching knowledge and efficacy—have been disputed in the coaching literature (18). One main criticism includes the low transferability of knowledge learned from coach education content to real-life coaching scenarios (19, 20). Indeed, different learning situations may uniquely influence the motivation to learn and the extent of the knowledge gained (9). Due to individual learning needs, unmediated and self-directed learning may be particularly appropriate and practical for youth sports coaches (21, 22). Further, with increasing evidence of the need for individualised educational content for coaches—which is reflective of their unique coaching contexts (1)—it has become imperative to investigate novel and innovative methods to enhance coach learning and inform facilitative coaching practice across a range of youth sports systems.

As the 21st century progresses and technology becomes inseparable from the human experience, the ability to harness technological advances to support coach learning becomes more essential. The current study aimed to investigate the use and perceptions of the *Coach Observation Tool* (COT), a self-directed mobile application designed to help coaches teach values and life skills in sports and physical education settings.

### 1.1 Innovations in coach education

Although youth sport is widely recognised as a valuable context to promote PYD, simply participating in sport does not guarantee the acquisition of values and life skills (23, 24). It is the intentional design and implementation of programmes, particularly by adult facilitators like coaches, who play a pivotal role in shaping youth's ability to learn and transfer these valuable skills (25). This emphasis on intentionality aligns with the growing recognition that while the inherent features and social dimensions of sport can contribute to the development of values and life skills through implicit learning, explicit approaches are crucial for maximising developmental gains (10, 26). However, many coaches face significant challenges in effectively adopting explicit values and life skills development approaches. One significant obstacle lies in the limitations of formal coach education programmes, which frequently fall short of adequately addressing the pedagogical knowledge and practical skills required for teaching values and life skills in sports (8). Formal coach education often prioritises technical and tactical aspects of coaching and neglects efforts to promote youth's healthy, holistic development (18, 27). Further compounding this challenge is the fact that many coaches are confronted with a variety of barriers, including time constraints and financial limitations, which inhibit coaches from seeking out educational opportunities (8).

One approach to coach education, which aims to provide coaches with the knowledge and skills to teach values and life skills, is using self-directed online learning programs. A viable and timely solution to bridge this gap in coach education and empower coaches to become more intentional in their programmatic decisions and practices. Self-administered online programmes offer coaches a valuable alternative to traditional, in-person training and workshops (8). Indeed, the flexibility of online platforms allows coaches to learn at their own pace and access a wide range of resources and expertise (21). Project SCORE represents a compelling example of a self-directed online learning programme. Through a series of modules that include reading materials, videos, and interactive activities, Project SCORE guides coaches in understanding the 4Cs framework (competence, confidence, connection, and character) and provides practical strategies for integrating these concepts into their coaching practices.

Findings from both Strachan et al. (22) and Ferreira et al. (21) suggest that Project SCORE is an effective self-directed learning tool for coaches, evidenced by its positive reception and impact on both coaches and athletes. For instance, coaches found the programme user-friendly and easily integrated into their existing practices, highlighting its accessibility and convenience. Findings from these studies further indicated that coaches valued the content, finding the material relevant and applicable to their unique coaching contexts. Moreover, Strachan et al. (28) demonstrated that coaches experienced personal growth through self-reflecting on their coaching styles, which, in turn, led to a deepened understanding of their role in fostering positive development. Yet, although Project SCORE has demonstrated the potential of self-directed online learning, research must continue to innovate coach education by investigating novel applications of self-directed learning.

### 1.2 Technological advances for unmediated and internal learning

The widespread use of technology (e.g., social media and mobile applications) as a form of unmediated learning has increasingly gained attention in recent decades (29, 30). In particular, systematic observation tools have been employed to understand coach behaviours in natural coaching contexts. For instance, Partington et al. (31) investigated the influence of technology (i.e., video feedback) on the behaviour of youth soccer coaches in the United Kingdom. Their study found that coaches discussed the positive impact of embracing technology, such as being prompted to self-reflect and become cognisant of differences between their beliefs and actions. Raya-Castellano et al. (32) similarly found video-based feedback to be a useful strategy to support willingness to change among coaches.

Technological advances have also occurred in response to the COVID-19 pandemic. For example, as a way in which to spur reflection, Moon et al. (33) developed a systematic observation instrument that can be used by coach learners during online simulations of sports activities and lessons. Further, Allan et al. (34) developed a systematic observation tool to examine explicit displays of emotions among coaches, including expressions of neutrality, happiness, affection, alertness, tenseness, anxiety, anger, and disappointment. Findings suggested that the tool enhanced personal insight and coach communication. Taken together, systematic observation tools can potentially support coach development [e.g., (35, 36)]. Specifically, the descriptive-analytical systems provide data on what coaches are doing and how this is aligned with coaches' practice intentions. Coaches can use the information for self-reflection or reflective conversations with knowledgeable others to enhance their understanding and development with practice and elicit significant behavioural change [e.g., (31, 37)]. However, the application of systematic observation to support coach development has remained limited due to reasons such as coaches' mindset in disregarding existing accumulated knowledge rather than considering ways to integrate new approaches with what is already known (38) or recognised operational issues, such as time-consuming coding and human error (39). As Trudel and Gilbert (40) alluded, technological advances greatly impact how coaches learn (e.g., personal learning work) to connect with others to share information or co-create knowledge deliberately. Hence, leveraging technology-based observations and feedback may provide a promising avenue for coaches to engage in self-directed learning, which may spur personal and professional development (41).

### 1.3 The present study

The present study explored the development of a coach observation tool (COT) mobile application to support coaches' learning and the participants' perceptions and recommendations of COT for future enhancement. This study was guided by four research questions: i) How can the COT be used to quantify coaches' efforts in promoting values and life skills? ii) Are there differences between the observed, planned and actual time coaches spend teaching values and life skills? iii) How do coaches use the feedback provided by the COT to guide their learning and future coaching practices? and iv) What are the coaches' recommendations to improve the COT?

### 1.4 Paradigmatic positioning

The mixed methods employed in the present study were guided by a pragmatic approach that uses research to investigate the practical implications of “real-world” problems that individuals experience (42). Pragmatism also acknowledges that reality is ever-changing and, thus, as a scientific paradigm, does not commit to one unique methodology or method to investigate individual experiences and perceptions (43). Rather, such a paradigm encourages the utility of multiple knowledge sources and forms of data to solve real-world problems. Given the purpose of the current study, to help coaches improve the integration of values and life skills into their practices, data were gleaned from both qualitative and quantitative sources.

### 1.5 The COT mobile application development and key features

Despite strong evidence highlighting that coaches must intentionally plan, deliver, and facilitate teaching values and life skills, there are limited tools to inform what happens during a coaching session. The Arizona State University Observation Instrument (ASUOI; 44) is a systematic observation tool that has been widely used to collect information on coach behaviours across different sports in both practices and competitions (45). However, issues with using the ASUOI include time-consuming coding and data collection. In light of these limitations, Koh et al. (39) adapted the ASUOI to measure the effects of a coach education course designed to help coaches foster values and life skills. Hence, changes were made to the original version of the ASUOI to quantify coaching behaviours associated with teaching values and life skills instead of simply focusing on sports skill development.

Overall, seven out of 14 categories (i.e., pre-instruction, concurrent instruction, post-instruction, questioning, positive modelling, negative modelling and praise) were revised to include two new subcategories, namely: “with values” (V) and “transfer” (T; see Table 1). For example, in a pre-instruction coach behaviour that includes the subcategory “With values,” the coach might say, “When learning the behind-the-back dribbling skill, it may be hard for some of you, but you've got to keep on trying even though you may experience multiple failures. Keep on trying and never give up is a show of determination.” “Transfer” refers to helping athletes make the connection between the values and life skills learned outside the sports context. For example, a pre-instruction coach behaviour that includes “With transfer” may include the following coach talking point:

**Table 1:** Modified ASUOI with subcategory descriptions.

Behavioural categories	Behavioural subcategories	Description
Use of first name		Using the first name or nickname when speaking directly to a player
Pre-instruction		*Initial Information given to player(s) preceding the desired action:*
	S	To be executed (S)
	V	Related to values (V)
	T	Related to values transfer (T)
Concurrent instruction		*Cues or reminders given during the actual:*
	S	Execution of the skill or play (S)
	V	Demonstration of the action/behaviour related to values (V)
	T	Demonstration of the action/behaviour related to values transfer (T)
Post-instruction		*Correction, re-explanation, or instructional feedback given after the:*
	S	Execution of the skill or play
	V	Demonstration of action/behaviour related to values
	T	Demonstration of action/behaviour related to values transfer
Questioning		*Any question to player(s) concerning:*
	S	Strategies, techniques, assignments, and so forth associated with the sport
	V	Values associated with the sport
	T	Values transfer associated with the sport
Physical assistance		Physically moving the player's body to the proper position or through the correct range of motion of a skill
Positive modelling		*A demonstration of the:*
	S	Correct performance of a skill or playing technique
	V	Desired behaviour/action related to values
	T	Desired behaviour/action related to values transfer
Negative modelling		*A demonstration of the:*
	S	Incorrect performance of a skill or playing technique
	V	Non-desired behaviour/action related to values
	T	Non-desired behaviour/action related to values transfer
Hustle		Verbal statement intended to intensify the efforts of the player(s)
Praise		*Verbal or non-verbal compliments, statements, or signs of acceptance related to:*
	S	Skill
	V	Values
	T	Values transfer
Scold		Verbal or non-verbal behaviours of displeasure
Management		Verbal statements related to organisational details of practice sessions not referring to strategies or fundamentals of the sport
Silence		Periods of time when the subject is not talking
Un**-**codable		Any behaviour that cannot be seen or heard or does not fit into the categories above

S refers to skills or strategies performed or to be performed. V is defined as instruction that includes values teaching, and T is defined as instruction that discusses how values may be transferred.

Similarly, when you face difficulty in your studies e.g., class assignment or test, you should keep trying or find solutions—approach your friends or teachers for help, and don't give up. This is a show of determination! Remember, the determination you show on the court—keep trying to get the behind-the-back dribbling skill right, and constantly improving, is the same determination you'll need to tackle obstacles outside of sports.

Five experts in sports coaching from Canada, Hong Kong and the United States ensured that the modified ASUOI maintained ecological validity. A pilot test was also conducted with coaches (*n* = 27) from team sports (*n* = 10; e.g., soccer, handball, and rugby), and individual sports (*n* = 17; e.g., swimming, taekwondo, and squash). Most coaches were male (*n* = 23, 85.2%) and in their 20s (*n* = 14, 51.9%). Fourteen coaches were placed under the intervention group who had undergone the Values and Principles in Sport (VPS) coach education programme and those who had not were placed in the control group (39). The VPS is a six-hour coach education course organised by Sport Singapore to train coaches in promoting values and life skills. Participant coaches must pass this course before coaching in any government school across Singapore. Two coaching sessions delivered by the participants were video recorded—one before the VPS course commenced and another two months after the course. The control group coaches were asked to record their two coaching sessions during the same period. 54 videos (ranging from 70–90 min) were recorded. The COT mobile application was used to code these coaching videos. Two trained research assistants coded all 54 videos. Intra-coder validity and inter-coder reliability for the coders were achieved (>85% of agreement).

**Figure 1:**
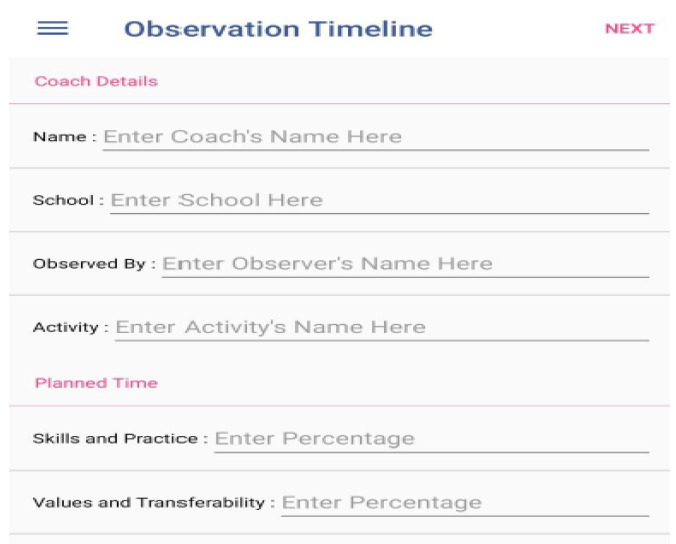
Observation details.

Before using the COT application to record coaching behaviours, details concerning the session (i.e., coach's name, school, observer, activity) must be registered. The application also requires users to enter the percentage of time in their lesson plans in which they plan to teach values and life skills. This information is used later for comparison between planned activities and actual coded activities (see Figure 1).

**Figure 2:**
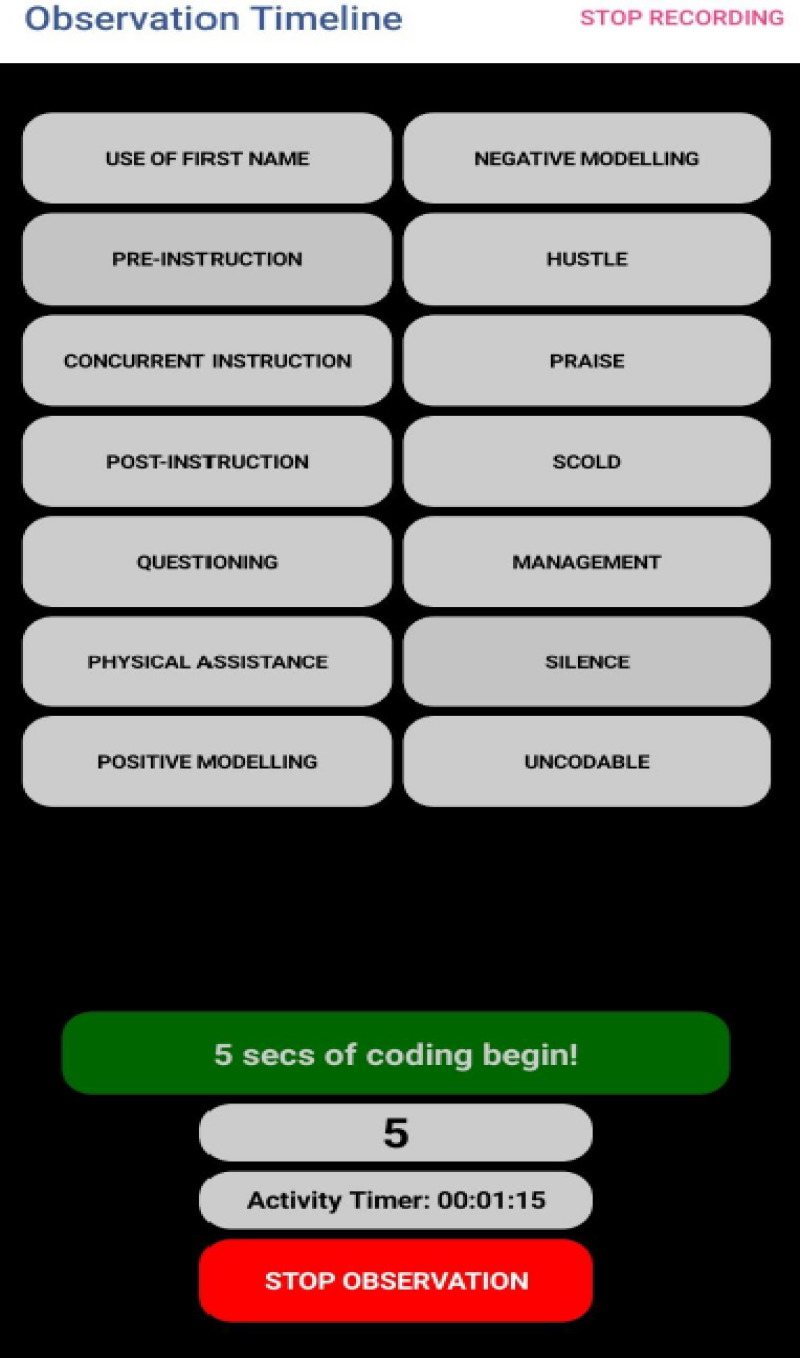
Observation timeline.

To use the COT, observers must enable access to the mobile phone camera and point it at the live sports activity being observed to shoot, record, and code *in situ*. Alternatively, recorded footage of lessons can be reviewed while using the COT for coding. A five-second observation interval is followed by a five-second coding interval where the categories from the ASUOI appear, requiring observers to decide on the category of the behaviour by tapping on the relevant label/button. For seven out of the total behaviours, a second-tier selection must be made from three additional categories consisting of “behaviour with values”, “behaviour without values” and “transfer” (see Figure 2).

**Figure 3:**
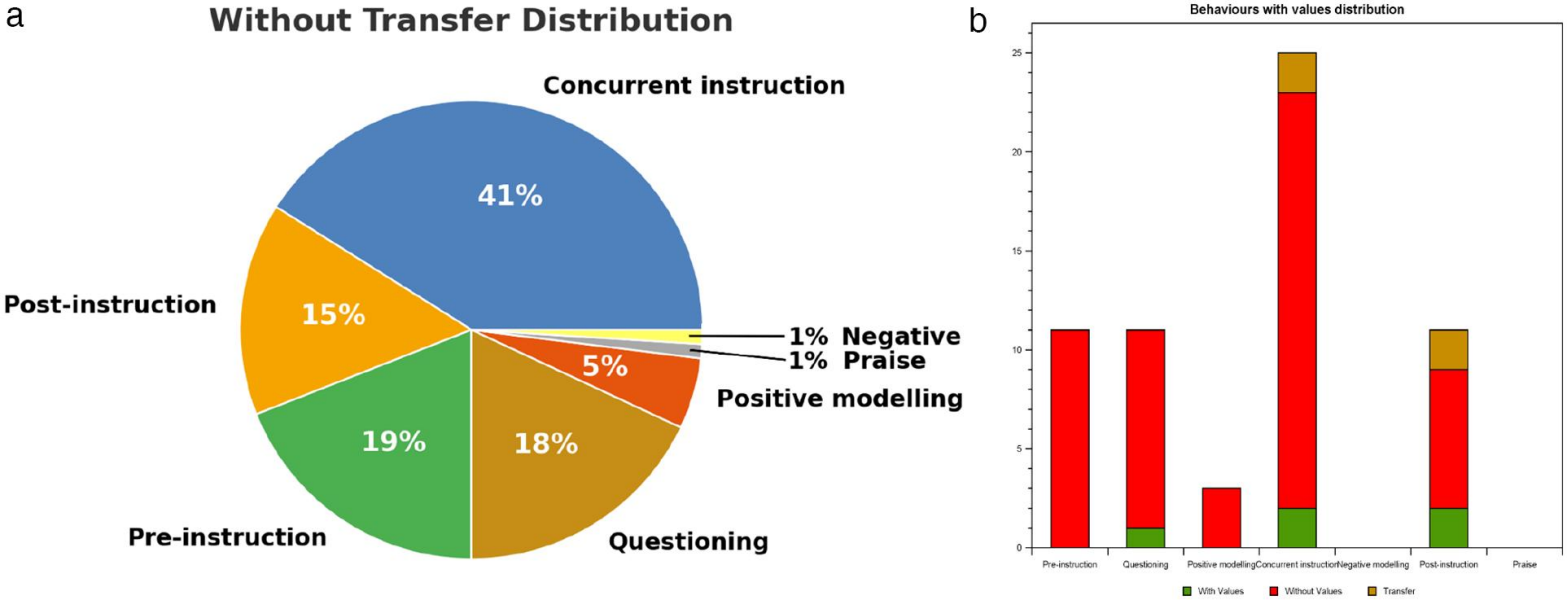
**(a)** Percentage of overall coaching behaviours without transfer distribution. **(b)** Coaching behaviours with values/life skills distribution and transfer.

One important advancement of this tool from the original ASUOI is automating manual coding and data analysis to minimise human errors with higher accuracy and reliability. The COT application also has a recording function with a timestamp to capture “critical moments” (i.e., coach behaviour that is useful for post-coaching/observation feedback and discussion). When coding is completed, the coded observations and the corresponding video recording will be shown on a timeline. The “statistics” function provides users access to the visualised data of the coaching behaviours. Five types of reports are then available, namely: i) the overall percentage distribution of the 14 coaching behaviours visualised in a chart; ii) the distribution of behaviours with values, without values and with transfer, for each of the seven modified behaviours, shown as a chart; iii) the overall distribution of behaviours with values; iv) the distribution of behaviours with transfer; and v) the distribution of behaviours without transfer from the seven modified behaviours (see Figures 3a,b).

A comparison can then be made between the observed and planned percentage of values and transfer-related coaching behaviours so that coaches/teachers are more aware of their intentionality in teaching values and life skills and reflect on how their coaching practice can be improved. A sharing function enables report-sharing with colleagues and mentors for further discussions to enhance awareness and encourage deeper reflection. Collaborating with a mentor can also draw attention to variables that may have been overlooked.

## 2 Methods

### 2.1 Participants

A total of 34 participants across the team and individual sports were involved in the study (*M* = 39.2, *SD* *=* 6.32). Fifteen of them taught at primary schools, and 19 taught at high schools/colleges. Twenty-nine participants were teacher coaches (23 males), and five were sports coaches (3 males). They had over six years of teaching/coaching experience (*M* = 12.3, *SD* = 7.42).

### 2.2 Data collection

Before data collection, ethics clearance was obtained from the first author's university ethical review board (IRB-2021-02-021). The participants were recruited using a convenience sampling technique. The first author contacted the potential participants through email or telephone to explain the purpose of the study and the degree of their involvement. Before and during the study, participants were reminded that their involvement in this study was voluntary and confidential and that they could withdraw at any time. Before data collection, a signed informed consent form was collected from all participants.

#### 2.2.1 Lesson plan

The participants were told to submit a 60 min coaching plan at least two days before the observation, stating the estimated amount of time (in percentage) they would develop sports skills, as well as values and life skills. They were informed of the focus of the observation—teaching values and life skills. Examining coaching plans aimed at understanding the alignment between planned and actual efforts to foster sports skills, values, and life skills.

#### 2.2.2 Video recordings

A research assistant video-recorded a total of 68 coaching sessions. The camera was positioned in a way that captured the whole coaching session without obstructing practice. The coach also had a wireless microphone to ensure verbal instructions were clearly captured for coding purposes. The same procedure was repeated one week later.

#### 2.2.3 The COT mobile application

The COT mobile application was used to code and quantify the participants' behaviours during lessons. Two trained research assistants familiar with the COT application coded all the videos. Both intra- and inter-coder reliability testing was conducted before coding the actual coaching videos.

After coding, a simple report was generated by the COT application and given to each participant within two days after the observation. The same procedure was repeated a week later for the second observation.

#### 2.2.4 Semi-structured interviews

The participants partook in a semi-structured interview immediately after the second observation. The interview aimed to gain insights into their coaching philosophy and strategies employed, and challenges faced in teaching values. Some questions include “In your PE/sports lessons, what is your main focus—skills vs. values? Why?” “Can you briefly share the time distribution for values and sports skills development of a typical session?” “How do you implement sports skills and values in your students/athletes? Provide concrete examples?” “Overall, do you feel conﬁdent in your ability to teach values?” “Do you face any challenges in teaching values and skills? If yes, what are those challenges?” “What factors would increase your confidence to teach values and skills?” Questions that aimed to understand participants' experiences in using the COT application include “Describe the type of support you received (e.g., using COT and the information provided) to plan and teach values and life skills” “What other support (features in COT) would you like to see to help you inculcate values and life skills in your teaching/coaching?”

### 2.3 Data analysis

Quantitative data collected from the COT were analysed using a paired-sample *t*-test to determine if there were differences between the planned and actual time spent on sports skills, as well as values and life skills across the two observations. Interview transcripts were also analysed using inductive thematic analysis (46) as it allows the researchers to centre their efforts on interpreting the data. The analysis was led by the two research assistants involved in the project. The inductive thematic analysis (46) consisted of five steps: i) the research assistants familiarised themselves with the data by reading the transcripts multiple times to identify initial meanings across the dataset; ii) codes were developed and refined into themes; iii) these initial themes were refined into the final set of themes; iv) a thematic table was created to organise themes; and v) quotes were selected to illustrate each theme.

To ensure rigour, the first author acted as a critical friend to the research assistants. He reviewed the codes and themes analysed by the research assistants and engaged in many rounds of discussion with them to encourage reflexivity and explore alternative explanations and interpretations of the data before reaching a consensus. These discussions focused on challenging the research assistants' assumptions, exploring potential biases, and examining the data from multiple theoretical perspectives. The credibility of the research assistants was established due to their experience in conducting qualitative interviews, the rigorous application of the thematic analysis method, and their transparent reporting of the analysis process.

## 3 Results

### 3.1 Quantitative

Quantitative results suggest that for both coaching sessions observed, participants' planned time was significantly greater than the actual time spent teaching values and life skills (*p* < .001 for both) as shown in Table 2.

**Table 2:** Planned vs. actual time on the development values.

Comparison	Mean difference between planned and actual time	*t*	Sig.
Planned values—Actual values (Session 1)	10.2	4.29	<.001
Planned values—Actual values (Session 2)	9.09	4.38	<.001

### 3.2 Qualitative

#### 3.2.1 Focus on sports skills

Overall, interview results aligned with the quantitative findings –coaches in the present study mainly focused on sports skills development. Although they focused on values and life skills in their plans, coaches hardly spent time teaching them. For instance, T5 mentioned that he only focuses on sports skills because “The content [in a training session] is still the main thing [to cover] …although I may try to plan about 60% (sports skills) and 40% (values)…getting athletes proficient in sports skills is still my priority…” Another participant mentioned that “while relevant values are incorporated into my lessons where possible, majority of the time I will still [focus] on skills development as there is a greater emphasis to cover the required curriculum.” (T18).

No participants mentioned prioritising values over sports skills development in their teaching practice:

… other than meeting the department outcomes… teaching outcomes…I think it's also because it's (sport skill) more tangible, it's more evident quickly… For the values part… it takes time… to pick up the value for them to learn, internalise, and for the teacher to observe the value… you want to focus on something that you can attain in a shorter time…

It is obvious that, due to curriculum requirements, coaches focused more on sports skills rather than integrating them with values and life skills development.

#### 3.2.2 Challenges of integrating values in lessons

Despite knowing the importance of through sports and physical education lessons, the participants believed this would reduce athletes' learning time: “The focus (of my lesson) is on their playtime. But of course, whenever there is a teachable moment, I will definitely stop and… educate my athletes on the values they should have” (T7).

Many coaches shared that they did not have proper training in this area. They mainly relied on their knowledge and used teachable moments instead of intentionally incorporating values and life skills into their lessons. They also believed that sports are a “movement” subject, so the focus should be on sports skill development. To them, teaching values and life skills should only happen when there is a “teachable moment.”

If there is some incident that has happened or some reason where a key value must be highlighted, then I will totally flip it around and then I'll do 70% value instead of 30% running… if it's a case of dishonesty in the team maybe in the past, which hasn't happened… if there's a need to address, then I'll modify (C5).

Similarly, T21 shared that,

when certain moments pop up, it brings out certain teachable moments… for example, if they are one man down, then there's some form of frustration, or there's some form of lack of coverage; those are things we talk about and unpack.

As such, although efforts can be made in planning lessons, unexpected situations can shift lesson focus. These situations were deemed challenging by coaches. Additionally, some values and life skills can take time to incorporate into lessons, and hence, using teachable moments and support from other teachers to brainstorm ideas would be beneficial, as illustrated by TC20:

sometimes, you need ideas to teach certain other values, like maybe compassion. It's quite hard to…put them towards. Certain values are hard to put into the lesson plan itself, but certain other values are easier to put in, so sometimes we need ideas from our peers to do it.

T9 highlighted another example,

While we do consider the affective domain [values] in our planning, but specifically… [we receive] little support… because we see that the values… can come along as teachable moments during the lesson itself… there are still many other avenues that we can tap into teaching values… and because of time constraints and everything else… we do not intentionally… plan the lessons in such a way that they revolve around the specific values we want to teach.

#### 3.2.3 Valuable data for feedback and reflection

All the participants recognised that having a report that provides information about their coaching practice would be useful for planning intentionally and teaching values and life skills. Specifically, the simple yet useful statistics generated through the COT application can provide concrete information and tangible feedback to facilitate quality reflection on their lessons:

When you're teaching … you're not observing yourself. So, if there's a peer or something…that can tell me … I've taught values this much today, or today, I totally never teach value … that feedback will… help me or could tell me, okay I have not been doing this, or I’ve been doing only this much. I need to do a bit more (T7).

#### 3.2.4 Increased awareness of teaching values and life skills

Through the outputs generated by the COT application, participants believed that they became more aware of their efforts toward teaching values and life skills:

My [teaching]chart… it really tells me about what happened in the lesson… it made me reflect on… how was my lesson? … I plan a lesson, but I may not have planned the values component to be very… specific… So that made me think…what can I do next, at the next lesson, to make it better? (T3)

Similarly, T12 shared that the information generated by the application serves as objective data concerning specific coaching outcomes. The information can potentially help them reflect critically on their teaching approach towards values and life skills and enhance future practice to benefit their athletes:

I think I have been teaching a lot of values to the kids but from the pie chart, I see I focus a lot on the [sports] skill set itself… so after I saw that I tried to bring up more scenarios where students…their values are being questioned… for example if you were to play a floorball game, a 3v3, then I will purposely… take out one player so it becomes a 3v2 then when the player with the three players… score a goal, then I will ask them why they score a goal… I will ask the other two players how you feel… it does happen in a game and somebody's injured and out for a while… So, I tried to create this kind of situation… it does take a little bit away from the skill… but… they still learn…

#### 3.2.5 Facilitates quality discussion

T11 provided the following example to illustrate how the COT application facilitated quality reflection and discussion on teaching and learning values and life skills explicitly:

we had a discussion… it's quite evident that we have cover values, and we do… know what we are supposed to do with the values and skills…but … maybe we're not explicit enough. So, these are things that we thought we could work on.

This aligned with T16'sclaims, which highlighted that the results generated through the COT application facilitate sharing and discussion among coaches, encouraging them to explore ways to improve their coaching knowledge:

“I think that pie chart was really very, very useful… I was able to discuss the lessons with other teachers in school… comparing… how much time I spent on doing everything…and seeking advice.”

#### 3.2.6 Limitations of the COT application

##### 3.2.6.1 Accuracy

While the participants acknowledged the numerous benefits of the COT application, they also highlighted a few concerns about the tool's accuracy because of the low number of observations conducted. They suggested using the application across more lessons to identify coaching trends:

[the app is] useful, but I don't think it's very accurate…and it's unfair for teachers just based on the two sessions. So, if we want to use that tool [to measure effectiveness], it should be across 10, 15, 20…lessons…And then we average it out, and it will be more accurate… (T11)

T16 and T9 also reinforced these notions: “The results only reflect on just one lesson out of many, many lessons… maybe this is just one lesson that I really don't have any value or don't have time to talk about” and,

[while the tool] can be useful, it also to a certain extent [depends] on the objective and the approach that is being used for the lesson itself because…the lesson that [the research team] came down for, it was mainly on self-assessment through video performance and through pair work… So, in terms of emphasis on values, it could also be on the lower side.

##### 3.2.6.2 Knowledge translation devices

The participants stated that changes should be made to the COT to better describe its outputs and the inherent implications for practice. For example, T19 shared that the report provided by the application is too brief and some do not understand the statistics and hence are unable to use them to enhance their coaching knowledge and practice:

[the report] is something brief… I would prefer something more detailed, because… I see this pie chart. I see the percentage allocation of behaviour…but I don't understand the stats behind it…and how come this one is transferable? That one's not transferable? …it does give me an idea… how much positive modelling I'm doing or negative? How much time did I spend on management? …so, it gives me an overview of how much time I'm spending… that makes me more aware because otherwise, I wouldn't know.

Similarly, T13 shared that besides providing statistics, support for interpreting the results and clarifying doubts coaches may have been essential moving forward: “When you explained to us, then [the app] becomes useful, but when you give [the report] to us, without explanation… it brought up more questions than… answers.”

C3 also suggested that breaking down the statistics would be ideal:

[the app] gives me more information about how my lesson went… which area do I need to focus on… but… it would be good, if you could provide us with a breakdown of what each behaviour means…and how can we use this data to enhance our teaching a bit better?

##### 3.2.6.3 Lack of consideration of athlete outcomes and feedback

Although the COT application provides useful information about the quality of participants' teaching, most of them felt the need for information concerning athletes' learning outcomes for a more balanced evaluation of their coaching effectiveness:

…what is more useful is to see whether it creates an outcome in the student in terms of their learning [of values] because, at the end of the day, what we want to see is a better character, which will lead to better student outcomes (T5).

T4 shared a similar sentiment and highlighted the importance of considering athletes' feedback about coaching practice, saying:

I would like to find out from the individual students' perspective … was this way of teaching effective? …They come to my class… they want to exercise… if we can evaluate them through this kind of teaching and learning scenario, I think it'd be good.

## 4 Discussion

The present study aimed to explore the development of a systematic observation tool (COT)—a mobile application and use it to support coach learning. Overall, the qualitative findings support the quantitative ones (i.e., coaches often planned to teach life skills but presented a scarcity of coaching practices with this purpose). While participants tried to teach values and life skills across lessons, sports skill development was still the main focus as it was a curricular demand set by governing bodies. Previous studies supported that practitioners lack the necessary skills and strategies to foster values and life skills in their practices (11, 47). This is the case because some coaches position teaching values and life skills as time-consuming and less relevant than sport skill development (27). Thus, combining these factors may have discouraged coaches from explicitly and systematically focusing on teaching values and life skills (8).

Youth sports coaches are recognised as the most meaningful sources of support for youth athletes to develop values and life skills (15). However, the priority placed on sports skills as the main content of coaching practice suggests a systemic issue that underscores the importance of transformative interactions between the coach and the athletes. The responses provided by the participants (e.g., C5 and T21) suggest that inter- and intrapersonal issues affect coaches' beliefs that sports skills, values, and life skills can co-exist and be integrated seamlessly into their lessons to benefit athletes. Unfortunately, coach education programmes often lack pedagogy and content related to values-driven coaching practices (11). Yet, research has shown that prior training in developing sports skills and tactics, life skills development, and mental health predicted higher satisfaction levels as a coach (48). Hence, a targeted approach for a coach education programme or having a dedicated team to specialise in values and life skills education and/or support stakeholders who are involved in sports is essential to plan and teach it effectively.

### 4.1 Facilitators using the COT mobile application

The participants in the present study reported that the COT application helped them to be more aware of their coaching behaviours, particularly concerning values and life skills. The observations conducted provided useful feedback in encouraging critical reflection at the individual level and facilitating quality discussions between coaches, their peers, and mentors. Indeed, past studies have shown that critical and in-depth discussions using quantitative data can improve coaches' awareness of their behaviours (32, 37). It also allows coaches to challenge each other's ideas and prompt critical reflection and critical action (15). In this case, peers and mentors could be the “critical friends” who encourage coaches to become more explicit in teaching values and life skills. As argued by Cope et al. (35), systematic observation tools can significantly contribute to a better understanding of coaching practice. Using the COT can provide a convenient way to obtain feedback and facilitate reflection among coaches. Indeed, reflection can promote self-awareness (49, 50) and help coaches engage in reflective practice (32).

Coaches in the present study might feel that deliberate and explicit teaching of values and life skills seemed too “unnatural”; hence, they might be reluctant to incorporate them into their coaching. To address the issues raised, there is an urgency for all decision-makers involved in sports programmes to give more emphasis and recognition to coaches who are fostering values and skills, making these objectives culturally relevant. Coach developers and programmers may need to support coaches, providing them more *autonomy* and *freedom* to decide on *what*, *when*, and *how* to *learn* values and life skills intentionally. Another point is that coaches may need more time to internalise and change their approaches towards teaching values and life skills. To do so, sports organisations can help coaches connect with others to share information and co-create knowledge meaningfully through *social learning spaces* and *communities of practice* (40).

For coaches to benefit from the information provided by the COT application, it is essential to provide specific and easy-to-understand information to guide them to reflect and suggest ways to enhance future practices. For example, specific pointers on the strengths and growth areas should be provided based on the data collected by the coaches concerned. In addition, relevant online resources could be offered to them if they decide to act on the growth areas. Such approaches support independent and targeted learning for coaches serious about their learning and development through searching for new information (40).

### 4.2 Barriers to using the COT mobile application

About 20% of the participants highlighted that using the COT application depends on a given lesson's objective. As lesson content varies weekly, it raises questions about the accuracy and the fairness of using the COT application in evaluating coaching effectiveness in terms of values and life skills development, and transfer of learning. Different lesson goals, such as teaching technical skills vs. teaching values and life skills, may require different coaching behaviours, which the application might not adequately capture. This could result in an evaluation that overlooks key aspects of coaching, especially those related to intangible outcomes like teamwork, resilience, or leadership.

About 8% of participants highlighted athletes' feedback as an important component in helping coaches decide on areas for improvement, which is consistent with past studies [e.g., (51)]. Similarly, Côtè and Gilbert (7) suggest that athlete feedback is crucial for evaluating a coach's effectiveness in values and life skills development, as it serves as a key indicator of the athlete's ability to demonstrate and articulate good character. Therefore, incorporating athlete perspectives allows coaches to track their athletes' responses, facilitating reflection and improvement of future lessons to positively impact their development.

Participants also suggested making the COT application more user-friendly by clearly explaining the information provided to the users. For example, specific strengths and areas for growth of the session, and the relevant resources that can help address the areas identified by the application. Specifically, if the growth area highlighted lacks intentionality, then making useful coaching tips and resources available is critical to support coach learning, including short instructional videos on i) identifying the values and life skills that can be integrated into the sports activities, ii) defining the meanings and observable behaviours, and iii) identifying the planned teachable moments and facilitating the learning and transfer during and at the end of the lesson. In this way, coaches have a clearer understanding of the data generated and can use the recommended tips and resources more effectively to enhance their introspection and reflection to facilitate abstract conceptualisation for effective planning and lesson enactment for the next lesson.

While the COT may be a promising tool to facilitate self-directed learning and enhance coaching knowledge factors such as accessibility, technological literacy, and applicability across diverse coaching environments (40) must be addressed to improve its reach and relevance.

### 4.3 Limitations and future research

The findings from the present study contributed to the literature by providing insights into how a systematic observation mobile application can be used to support coach learning. However, some limitations must be considered.

First, only two sessions were observed per coach, which may limit the reliability of the findings as changing coaching behaviours requires time. Future studies could include longitudinal data and a higher number of observations to determine the usefulness of the COT in promoting self-directed coach learning.

Second, the lesson observation requires manual coding through the application, which can be time and labour-consuming. This may hinder coaches' motivation to use the COT to inform their coaching practices and support their learning. Furthermore, it may be challenging to use the application if the coder is not clear about the categories and definitions of each coaching behaviour of the application (i.e., reliability issues). As Stumbo and McWalters (52) highlighted, it is important to ensure that the outcomes of the evaluation process are reliable, objective, and of good quality. Future studies could explore ways to streamline the manual coding process in the application, reducing the time and labour involved to enhance coaches' motivation to use the tool. Additionally, future research could investigate methods to improve clarity around the categories and definitions of coaching behaviours to address potential reliability issues.

Third, the short observation and coding window of five seconds, which occurs in consecutive intervals, may create inaccuracies in the coding as the coding/decision interval runs over to the following observation interval. Future applications can consider coding behavioural changes of coaches (i.e., event-based coding) instead of time intervals to understand the link between each coaching episode better. For example, how the athletes respond to the decisions made by their coaches to understand and advance the quality of their decision-making in teaching and learning. Thus, future studies can explore modifications to the application to shift from time-based intervals to coding behavioural changes across coaching episodes. This change could provide a more accurate understanding of the link between coaching decisions and athlete responses, offering valuable insights into the quality of decision-making in teaching and learning.

Fourth, it is crucial to consider various factors that may impact coaches' teaching approach, such as the age and experience of the athletes. For example, younger athletes may learn best if coaches use explicit strategies when compared to adolescents who may prefer indirect ones (53). The differences in strategies used to cater to different age groups of athletes (39) may, therefore, affect the categories identified by the COT application, which may further affect the data generated from each category. Hence, it might be useful for further studies to consider athletes' responses and age groups, which might impact the time spent teaching values and life skills in the sports and physical education (SPE) settings.

Fifth, athlete data can provide insights into how youth experience and respond to coaching practices that aim to foster values and life skills. Future versions of the COT could include athlete feedback with particular emphasis on how youth learn values and life skills, as well as their wants and needs.

Finally, exploring how social, cultural and political variables influence coaches' perceptions of values and life skills could significantly enhance the discussion about the reach of the COT. Given that sports coaching is deeply influenced by sociocultural contexts, a comparative analysis concerning the effectiveness of the COT across different countries may be beneficial to advance this line of inquiry (54).

## 5 Conclusion

The results from the present study suggest that the COT application may be useful in facilitating coaches' reflections and reflexivity, increasing their awareness about values and life skills integration in their coaching practices. However, it is necessary to help them understand and use the information generated by the COT application to inform future lessons. There is also a need to enhance the COT application to collect athletes' perspectives besides mapping coaching behaviours to provide a complete overview of the coaching and learning environment. Findings from the present study provide preliminary evidence that the COT mobile application can be a useful tool to support “unmediated” learning for “deliberate self-development practitioners” (40), specifically in planning and delivering values and life skills education in their lessons.

Developing values and life skills through SPE requires time, repetitive behaviours, reinforcement, and continued support (39). Despite the labour-intensiveness of the mobile application and other technical limitations of the COT, this study provides valuable direction for practitioners and coach developers to consider using digital technology to support coach learning, enhance their knowledge, and advance their competency in coaching values and life skills in SPE and beyond.

## Data Availability

The raw data supporting the conclusions of this article will be made available by the authors, without undue reservation.

## References

[1] Newman TJ Ortega RM Lower LM Paluta LM. Informing priorities for coaching education: perspectives from youth sport leaders. Int J Sports Sci Coach. (2016) 11(3):436–45. 10.1177/1747954116645207

[2] Dorsch TE Smith AL Blazo JA Coakley J Côté J Wagstaff CR Toward an integrated understanding of the youth sport system. Res Q Exerc Sport. (2022) 93(1):105–19. 10.1080/02701367.2020.1810847 32960153

[3] Pierce S Gould D Camiré M. Definition and model of life skills transfer. Int Rev Sport Exerc Psychol. (2017) 10(1):186–211. 10.1080/1750984X.2016.1199727

[4] Chelladurai P. Leadership in sports: a review. Int J Sport Psychol. (1990) 21(4):328–54.

[5] Horn TS Lox C Labrador F. The self-fulfilling prophecy theory: when coaches’ expectations become reality. Appl Sport Psychol. (1993) 6:81–105.

[6] Smith RE Smoll FL Curtis B. Coach effectiveness training: a cognitive-behavioral approach to enhancing relationship skills in youth sport coaches. J Sport Exerc Psychol. (1979) 1(1):59–75. 10.1123/jsp.1.1.59

[7] Côté J Gilbert W. An integrative definition of coaching effectiveness and expertise. Int J Sports Sci Coach. (2009) 4(3):307–23. 10.1260/174795409789623

[8] Camiré M Newman TJ Bean C Strachan L. Reimagining positive youth development and life skills in sport through a social justice lens. J Appl Sport Psychol. (2022) 34(6):1058–76. 10.1080/10413200.2021.1958954

[9] Camiré M Santos F Newman T Vella S MacDonald DJ Milistetd M Positive youth development as a guiding framework in sport research: is it time to plan for a transition? Psychol Sport Exerc. (2023) 69:102505. 10.1016/j.psychsport.2023.102505 37665940

[10] Bean C Kramers S Forneris T Camiré M. The implicit/explicit continuum of life skills development and transfer. Quest. (2018) 70(4):456–70. 10.1080/00336297.2018.1451348

[11] Newman T Black S Santos F Jefka B Brennan N. Coaching the development and transfer of life skills: a scoping review of facilitative coaching practices in youth sports. Int Rev Sport Exerc Psychol. (2023) 16(1):619–56. 10.1080/1750984X.2021.1910977

[12] Halstead JM Taylor MJ. Learning and teaching about values: a review of recent research. Camb J Educ. (2000) 30(2):169–202. 10.1080/713657146

[13] Gould D Carson S. Life skills development through sport: current status and future directions. Int Rev Sport Exerc Psychol. (2008) 1(1):58–78. 10.1080/17509840701834573

[14] Stelter R. The coach as a fellow human companion. In: van Zyl L Stander M Odendaal A, editors. Coaching Psychology: Meta-Theoretical Perspectives and Applications in Multicultural Contexts. Cham: Springer (2016). p. 47–66. 10.1007/978-3-319-31012-1_3

[15] Newman TJ Santos F Cardoso A Pereira P. The experiential nature of coach education within a positive youth development perspective: implications for practice and research. Int Sport Coach J. (2020) 7(3):398–406. 10.1123/iscj.2019-0106

[16] Dewey J. Experience and Education. New York, NY: Simon and Schuster (1938).

[17] Werthner P Trudel P. A new theoretical perspective for understanding how coaches learn to coach. Sport Psychol. (2006) 20(2):198–212. 10.1123/tsp.20.2.198

[18] Santos F Gould D Strachan L. Research on positive youth development-focused coach education programs: future pathways and applications. Int Sport Coach J. (2019) 6(1):132–8. 10.1123/iscj.2018-0013

[19] Nelson L Cushion C Potrac P. Enhancing the provision of coach education: the recommendations of UK coaching practitioners. Phys Educ Sport Pedagogy. (2013) 18(2):204–18. 10.1080/17408989.2011.649725

[20] Koh KT Mallett CJ Camire M Wang CKJ. A guided reflection intervention for high-performance basketball coaches. Int Sport Coach J. (2015) 2(3):273–84.

[21] Ferreira M Santos F Fernández-Villarino MA Mergler J Strachan L MacDonald DJ. Delivering project SCORE in competitive youth sport settings. Front Sports Act Living. (2024 ) 6:1439822. 10.3389/fspor.2024.1439822 39229249 PMC11368764

[22] Strachan L MacDonald DJ Côté J. Project SCORE! coaches’ perceptions of an online tool to promote positive youth development in sport. Int J Sports Sci Coach. (2016) 11(1):108–15. 10.1177/1747954115624827

[23] Ault KJ Blanton JE Pierce S. Student-athletes’ perceptions of relationship quality and life skills development. J Appl Sport Psychol. (2024) 36(1):139–60. 10.1080/10413200.2023.2197970

[24] Bean C Forneris T. Is life skill development a by-product of sport participation? perceptions of youth sport coaches. J Appl Sport Psychol. (2017) 29(2):234–50. 10.1080/10413200.2016.1231723

[25] Camiré M Santos F. Promoting positive youth development and life skills in youth sport: challenges and opportunities amidst increased professionalization. J Sport Pedagog Res. (2019) 5(1):27–34.

[26] Holt NL Neely KC Slater LG Camiré M Côté J Fraser-Thomas J A grounded theory of positive youth development through sport based on results from a qualitative meta-study. Int Rev Sport Exerc Psychol. (2017) 10(1):1–49. 10.1080/1750984X.2016.1180704 27695511 PMC5020349

[27] Koh KT Camiré M Lim Regina SH Soon WS. Implementation of a values training program in physical education and sport: a follow-up study. Phys Educ Sport Pedagogy. (2017) 22(2):197–211. 10.1080/17408989.2016.1165194

[28] Strachan L McHugh TL Mason C. Understanding positive youth development in sport through the voices of indigenous youth. J Sport Exerc Psychol. (2018) 40(6):293–302. 10.1123/jsep.2018-0035 30517819

[29] Cope EJ Partington M Cushion CJ Harvey S. An investigation of top-level youth soccer coaches’ questioning practice. Qual Res Sport Exerc Health. (2016) 8:380–93. 10.1080/2159676X.2016.1157829

[30] Lefebvre J Turnnidge J Cote J. A systematic observation of coach leadership behaviors in youth sport. J Appl Sport Psychol. (2021) 33:377–86. 10.1080/10413200.2019.1609620

[31] Partington M Cushion CJ Cope E Harvey S. The impact of video feedback on professional youth football coaches’ reflection and practice behaviour: a longitudinal investigation of behaviour change. Refl Pract. (2015) 16(5):700–16. 10.1080/14623943.2015.10717

[32] Raya-Castellano PE Reeves MJ Fradua-Uriondo L McRobert AP. Post-match video-based feedback: a longitudinal work-based coach development program stimulating changes in coaches’ knowledge and understanding. Int J Sports Sci Coach. (2021) 16(6):1259–70. 10.1177/17479541211017276

[33] Moon J Webster CA Brian A Stodden DF Mulvey KL. Development of the system for observing virtual real time lessons in physical education (SOVRTL-PE): a tool to support preservice teachers’ applied learning experiences. Comput Educ. (2023) 196:104738. 10.1016/j.compedu.2023.104738

[34] Allan V Turnnidge J Vierimaa M Davis P Côté J. Development of the assessment of coach emotions systematic observation instrument: a tool to evaluate coaches’ emotions in the youth sport context. Int J Sports Sci Coach. (2016) 11(6):859–71. 10.1177/1747954116676113

[35] Cope E Cushion CJ Harvey S Partington M. Re-visiting systematic observation: a pedagogical tool to support coach learning and development. Front Sports Act Living. (2022) 4:962690. 10.3389/fspor.2022.962690 36081620 PMC9446450

[36] De Marco G Mancini V Wuest D. Reflections on change: a qualitative and quantitative analysis of a baseball coach’s behaviour. J Sport Behav. (1996) 20:135–63.

[37] Cope E Cushion CJ Harvey S Partington M. Investigating the impact of a Freirean informed coach education programme. Phys Educ Sport Pedagogy. (2021) 26(1):65–78. 10.1080/17408989.2020.1800619

[38] Cushion CJ. Applying game centred approaches in coaching: a critical analysis of the ‘dilemmas of practice’ impacting change. Sport Coach. (2013) 2:61–76. 10.1080/21640629.2013.861312

[39] Koh KT Komar J Tarkington N Tan ES. The effects of values and principles in sports coach education course designed to promote values-driven coaching styles. Int J Sports Sci Coach. (2024) 19(6):2272–85. 10.1177/17479541241266975

[40] Trudel P Gilbert W. Foundations and evolution of coach development. In: Rynne SB Mallett CJ, editors. The Routledge Handbook of Coach Development in Sport. 1st ed. New York, NY: Routledge (2024). p. 1–22. 10.4324/9781003160939

[41] Cushion CJ Townsend RC. Technology-enhanced learning in coaching: a review of literature. Educ Rev. (2019) 71(5):631–49. 10.1080/00131911.2018.1457010

[42] Poucher ZA Tamminen KA Caron JG Sweet SN. Thinking through and designing qualitative research studies: a focused mapping review of 30 years of qualitative research in sport psychology. Int Rev Sport Exerc Psychol. (2019) 13(1):163–86. 10.1080/1750984X.2019.1656276

[43] Kowalski RM Toth A Morgan M. Bullying and cyberbullying in adulthood and the workplace. J Soc Psychol. (2017) 158(1):64–81. 10.1080/00224545.2017.1302402 28402201

[44] Lacy AC Darst PW. Evolution of a systematic observation system: the ASU coaching observation instrument. J Teach Phys Educ. (1984) 3(3):59–66. 10.1123/jtpe.3.3.59

[45] Lacy AC Goldston PD. Behavior analysis of male and female coaches in high school girls’ basketball. J Sport Behav. (1990) 13(1):29. Available online at: https://api.semanticscholar.org/CorpusID:141801315

[46] Naeem M Ozuem W Howell K Ranfagni S. A step-by-step process of thematic analysis to develop a conceptual model in qualitative research. Int J Qual Methods. (2023) 22:16094069231205789. 10.1177/1609406923120578

[47] Kramers S Turgeon S Bean C Sabourin C Camiré M. Examining the roles of coaching experience and coach training on coaches’ perceived life skills teaching. Int J Sports Sci Coach. (2020) 15(4):576–83. 10.1177/1747954120922367

[48] Anderson-Butcher D Bates S Lo MT. Coach training participation and athlete life skill development: findings from the US national coach survey. Quest. (2024). 10.1080/00336297.2024.2407140

[49] Taylor S Werthner P Culver D Callary B. The importance of reflection for coaches in parasport. Refl Pract. (2015) 16(2):269–84. 10.1080/14623943.2015.1023274

[50] Whitehead AE Cropley B Huntley T Miles A Quayle L Knowles Z. ‘Think aloud’: toward a framework to facilitate reflective practice amongst rugby league coaches. Int Sport Coach J. (2016) 3(3):269–86. 10.1123/iscj.2016-0021

[51] Nasatir SI. The Effects of Consistent Observation and Feedback on Teacher Practice and Motivation to Refine Instruction. Available online at: https://digitalcommons.nl.edu/diss/173/ (Accessed May 18, 2025).

[52] Stumbo C McWalters P. Measuring effectiveness: what will it take? Educ Leadersh. (2011) 68(4):10–5. Available online at: https://www.ascd.org/el/articles/measuring-effectiveness-what-will-it-take

[53] Helwig CC Ryerson R Prencipe A. Children’s, adolescents’, and adults’ judgments and reasoning about different methods of teaching values. Cogn Dev. (2008) 23(1):119–35. 10.1016/j.cogdev.2007.06.003

[54] Borges M Rosado A Lobinger B Freitas F de Oliveira RF. The cross-cultural training needs of football coaches. Int Sport Coach J. (2023) 11(1):105–12. 10.1123/iscj.2022-0018

